# Phenotypic variability in gap junction syndromic skin disorders: experience from KID and Clouston syndromes’ clinical diagnostics

**DOI:** 10.1007/s13353-014-0266-1

**Published:** 2015-01-10

**Authors:** Anna Kutkowska-Kaźmierczak, Katarzyna Niepokój, Katarzyna Wertheim-Tysarowska, Aleksandra Giza, Maria Mordasewicz-Goliszewska, Jerzy Bal, Ewa Obersztyn

**Affiliations:** 1 Department of Medical Genetics, The Institute of Mother and Child, ul.Kasprzaka 17a, 01-211 Warsaw, Poland; 2 One Day Pediatric Department, The Institute of Mother and Child, Warsaw, Poland

**Keywords:** Keratitis–ichthyosis–deafness syndrome, KID, Hidrotic ectodermal dysplasia, Clouston syndrome, *GJB2*, *GJB6*

## Abstract

Connexins belong to the family of gap junction proteins which enable direct cell-to-cell communication by forming channels in adjacent cells. Mutations in connexin genes cause a variety of human diseases and, in a few cases, result in skin disorders. There are significant differences in the clinical picture of two rare autosomal dominant syndromes: keratitis–ichthyosis–deafness (KID) syndrome and hidrotic ectodermal dysplasia (Clouston syndrome), which are caused by *GJB2* and *GJB6* mutations, respectively. This is despite the fact that, in both cases, malfunctioning of the same family proteins and some overlapping clinical features (nail dystrophy, hair loss, and palmoplantar keratoderma) is observed. KID syndrome is characterized by progressive vascularizing keratitis, ichthyosiform erythrokeratoderma, and neurosensory hearing loss, whereas Clouston syndrome is characterized by nail dystrophy, hypotrichosis, and palmoplantar keratoderma. The present paper presents a Polish patient with sporadic KID syndrome caused by the mutation of p.Asp50Asn in *GJB2*. The patient encountered difficulties in obtaining a correct diagnosis. The other case presented is that of a family with Clouston syndrome (caused by p.Gly11Arg mutation in *GJB6*), who are the first reported patients of Polish origin suffering from this disorder. Phenotype diversity among patients with the same genotypes reported to date is also summarized. The conclusion is that proper diagnosis of these syndromes is still challenging and should always be followed by molecular verification.

## Introduction

Two rare congenital disorders, keratitis–ichthyosis–deafness (KID) syndrome [OMIM 148210] and Clouston syndrome (hidrotic ectodermal dysplasia 2) [OMIM 129500], are caused by mutations in genes coding connexin proteins (*GJB2* and *GJB6*, respectively), and both of them have skin manifestation.

Despite the malfunctioning of proteins from the same family and some overlapping of such clinical features as nail dystrophy, hair loss, and palmoplantar keratoderma, the clinical picture of these syndromes differs significantly.

KID syndrome is a very rare congenital autosomal dominant disorder of keratinization with abnormal differentiation of the epidermis and aberrant formation of the cornified layer. The illness is characterized by progressive vascularizing keratitis, ichthyosiform erythrokeratoderma, and neurosensory hearing loss.

Most patients with KID syndrome are sporadic; only about 100 have been reported so far. The syndrome was first described in 1915 by Frederick S. Burns in a 16-year-old boy with congenital atypical ichthyosiform erythrokeratoderma, palmoplantar keratosis, and sensorineural hearing loss (Burns 1915). The term “KID syndrome” was later introduced as an acronym of the first letters of the main clinical symptoms. There are, however, arguments that this acronym does not define the disorder precisely; for example, changes in the skin are not ichthyosis but ichthyosis-like erythrokeratoderma, and keratitis can be absent at the onset of the illness (Skinner et al. 1981).

Clouston syndrome, or hidrotic ectodermal dysplasia, is another autosomal dominant rare disorder characterized by nail dystrophy, hypotrichosis, and palmoplantar keratoderma. The clinical expression can vary, but nails are predominantly affected. They are thick, hyperplastic, and deformed with onycholysis. Hair is dry, fine, and brittle, and may be absent from the scalp, axillary, and pubic region. Moderate to severe hyperkeratosis is often present, with reduced keratinocytes desquamation (Kibar et al. 1996). Sweating and the teeth are normal (Hassed et al. 1996).

Two cases are described in the present paper: that of a Polish patient with sporadic KID syndrome who encountered difficulties in obtaining a correct diagnosis and a family with Clouston syndrome who are the first reported patients of Polish origin.

## Clinical reports

### Patient 1

The proband is the first and only child of nonconsanguineous parents. He was born at 35 weeks of gestation after pregnancy complicated by the mother’s hypertension and a herpes simplex infection. His birth weight was 2,630 g (25th–50th centile), length 51 cm (95th centile), head circumference 32 cm (25th–50th centile), the Apgar score was 6 points in the 1st minute and 8 points in the 5th minute. The audiologic screening after birth revealed deafness. He was noted to have generally hyperkeratotic skin, especially thick on the back, and congenital ichthyosis was suspected. In the second week, a very thick hyperkeratotic layer of the skin on the back was removed by the parents during his bath. Histopathologic investigation of skin biopsy did not confirm ichthyosis. The next suspected disease was Netherton syndrome, but the patient’s hair evaluation did not reveal bamboo hair, which is typical for this syndrome. Menkes syndrome was also considered in the differential diagnosis elsewhere.

**Fig 1:**
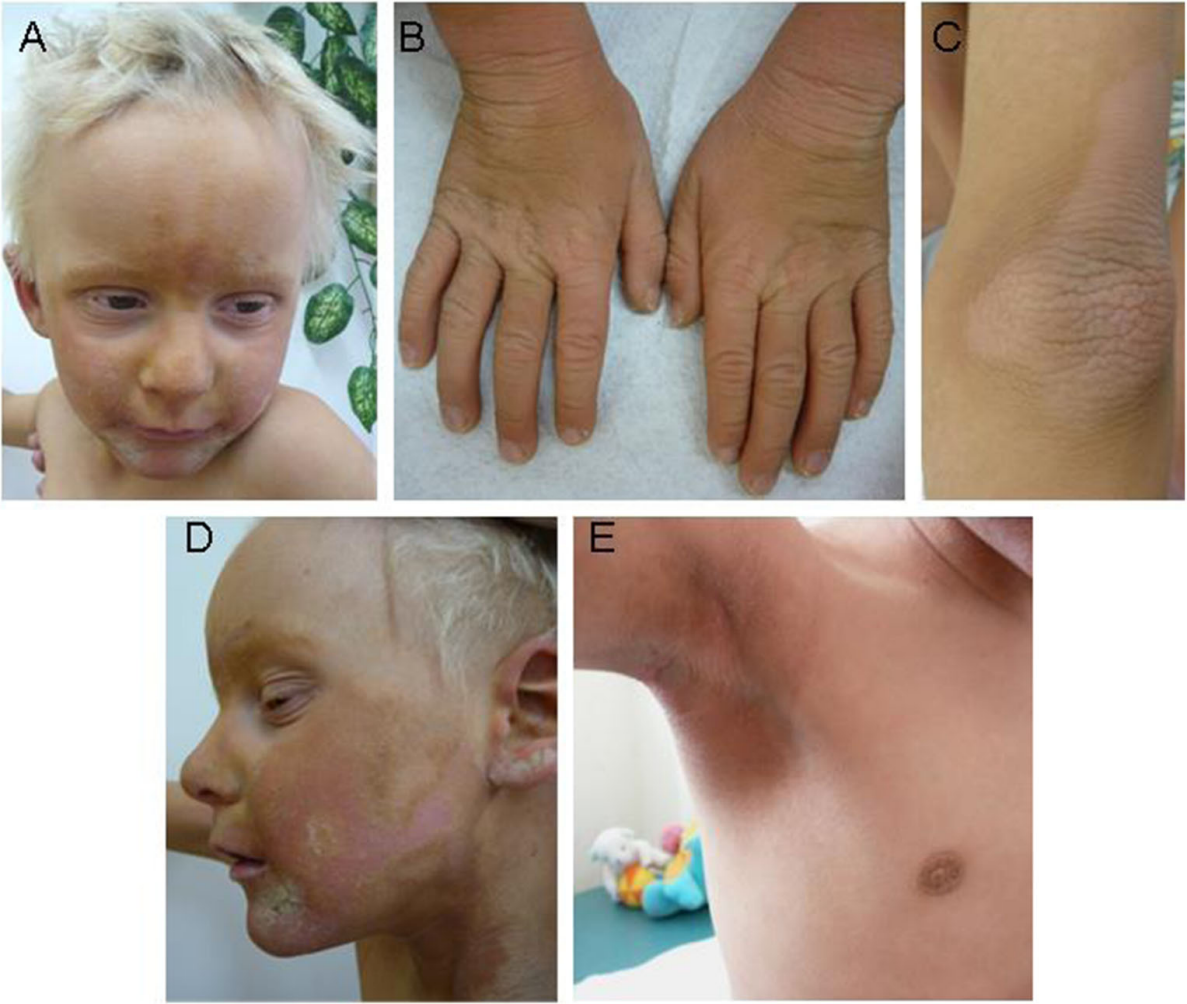
Phenotype characteristics of patient 1: **a**, **d** wart-like hyperkeratotic plaques on an erythematous base on the cheek, ears, and chin; **b** reticulated hyperkeratosis on the hands with normal nails; **c** hyperkeratotic skin over elbow; **e** acanthosis nigricans in the axillary region and around the nipple

The boy was examined for the first time in the Genetics Department of the Institute of Mother and Child in Warsaw (Poland) in the 4th year of his life (Fig. 1). He had hyperkeratotic skin all over the body but especially over his joints, reticulated hyperkeratosis on the hands and feet, with normal nails. Wart-like hyperkeratotic plaques on an erythematous base, which were initially interpreted as ichthyotic but, in fact, turned out to be ichthyosis-like erythrokeratoderma, were symmetrically located on the cheeks, ears, and chin. He also had deep furrows around the mouth and eyes. Eyebrows, eyelashes, and body hair were absent. His scalp hair was stiff and dry. Acanthosis nigricans in the axillary and inner elbow regions and also around the nipples was observed. The patient manifested sensorineural hearing loss, photophobia, hypohidrosis, and increased susceptibility to cutaneous infections. His dentition was regular. Ophthalmologic examination at the age of 5 years did not reveal keratitis. Somatic and psychomotor development was normal. The diagnosis of KID syndrome was suggested.

### Patient 2

**Fig. 2:**
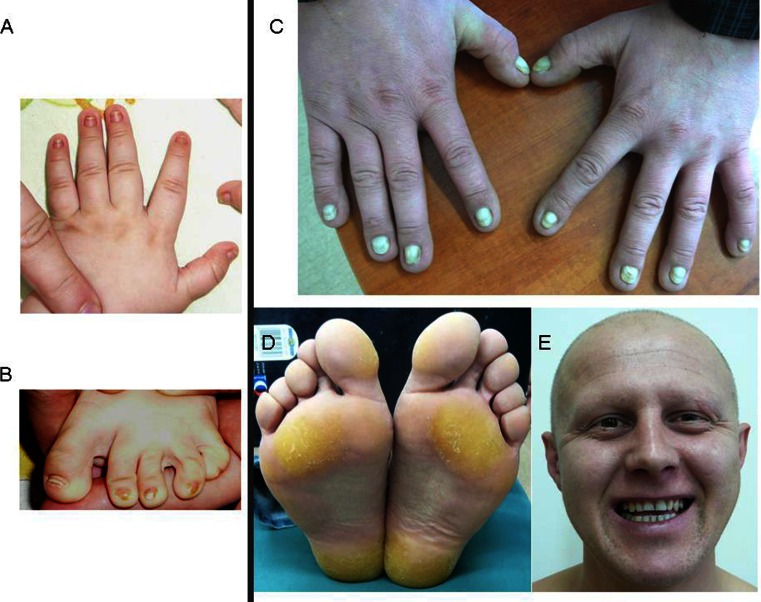
Phenotype characteristics of patients 2, **a**, **b** dysplastic nails and toes, and 3, **c** thickened, dysplastic nails, **d** plantar hyperkeratosis, **d** partial alopecia, sparse eyebrows and eyelashes, normal teeth

The proband is the first and only child of nonconsanguineous parents. He was born at 41 weeks of gestation after an uncomplicated pregnancy. His birth weight was 3,820 g (75th centile), length 56 cm (>95th centile), head circumference 32 cm (5th centile), the Apgar score was 9 points in the 1st minute. and 10 points in the 3rd minute. At the age of 8 months, he was admitted to the Genetic Counseling Department of the Institute of Mother and Child in Warsaw because of hypotonia and retardation in motor development. Physical examination showed dysplastic nails of the fingers and toes, but the hair, eyebrows, and eyelashes were normal (Fig. 2). His head circumference was within the normal range. Cerebral ultrasound at the age of 6 months was described as normal and the psychomotor development of this child at the age of 4 years was normal.

### Patient 3

**Fig. 3:**
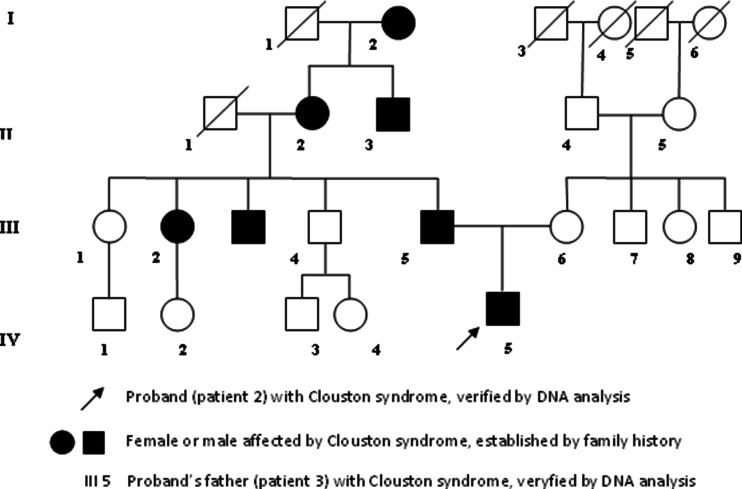
Pedigree of the family of patients 2 and 3 with Clouston syndrome. Affected males are represented with *filled squares* and affected females with *filled circles*

The patient was the 30-year-old father of patient 2 who had never been diagnosed previously. He had partial alopecia and sparse eyebrows and eyelashes. His nails were thickened and dysplastic, with subungual hyperkeratosis, severe curvature, and yellow discoloration. The skin of his palms and soles was hyperkeratotic and mildly hyperpigmented on the joints. Sweating and teeth were normal. The patient had normal eyebrows and eyelashes in his early childhood, and then he lost them. Progressive hair loss began after the age of 22 years. Several members of this family were also affected (his brother, mother, and uncle) (Fig. 3). His mother has similar signs, apart from hair, which was present (it was, however, always sparse). The diagnosis of familial Clouston syndrome was suggested.

## Methods

In the three patients described herein, DNA was isolated from peripheral blood leukocytes using Genomic Maxi AX (A&A Biotechnology). A mutation analysis based on Sanger sequencing of the coding exon of the *GJB2* gene was performed in patient 1 and of the *GJB6* gene in patients 2 and 3 (primer sequences available on request). Fluorochromatograms were analyzed in Mutation Surveyor software (SoftGenetics) using NM_004004.5 and NM_006783.4 as reference sequences. The parents of patients 1 and 2, and patient 3 gave informed consent to participate in the study and to publish the patient photographs.

## Results

The results of DNA analysis in the three patients confirmed the clinical diagnosis of KID syndrome in patient 1 [heterozygous mutation p.Asp50Asn (c.148G>A) in *GJB2*] and Clouston syndrome in patients 2 and 3 [mutation p.Gly11Arg (c.31G>C) in *GJB6*].

## Discussion

The present study describes patients with very rare genetic disorders caused by mutations in the connexin genes: KID syndrome caused by a de novo mutation p.Asp50Asn in the *GJB2* gene and familial Clouston syndrome caused by a mutation of p.Gly11Arg in the *GJB6* gene inherited from the proband’s father. The clinical manifestations of both syndromes vary, but manifestations in the skin and its appendages are predominant (Table 1) (Caceres-Rios et al. 1996).

**Table 1:** Diagnostic criteria for keratitis–ichthyosis–deafness (KID) syndrome (minor criteria may not be present) (Caceres-Rios et al. 1996)

**Major criteria**	
Erythrokeratoderma (100 %)	
Neurosensorial deafness (100 %)	
Vascularizing keratitis	
Reticulated palmoplantar hyperkeratosis	
Alopecia	
**Minor criteria**	
Susceptibility to infections	
Dental dysplasia	
Hypohidrosis	
Growth delay	

Skin lesions in KID syndrome can be seen from birth as red, dry, wrinkled skin with wart-like or hyperkeratotic plaques of erythrokeratoderma. The natural history of this syndrome is characterized by chronic bacterial and fungal skin and mucous membrane infections, with a sometimes fatal course in the first year of life (Gilliam and Williams 2002; Janecke et al. 2005) and later by a higher risk of squamous cell carcinoma (SCC) of the skin and oral mucosa (Hazen et al. 1989). Despite being the major symptoms in KID syndrome, erythrokeratoderma and deafness are also characteristic for the other disorder caused by mutations in genes coding connexin Cx31 (*GJB3*; OMIM 603324) and Cx30.3 (*GJB4*; OMIM 605425)—erythrokeratoderma variabilis of Mendes da Costa; however, keratitis is not part of this syndrome. In KID syndrome, keratitis and corneal vascularization may appear later in life (at the onset of puberty) and can progress to blindness (Gómez-Faiña et al. 2006). Moreover, KID syndrome is the only connexin disorder which is associated with a risk of at least 12 % that SCC can develop. An early diagnosis of this syndrome is, thus, essential to avoid irreversible eye damage and to support patients with regular oncological monitoring (Avshalumova et al. 2014).

Several mutations in the *GJB2* gene causing KID syndrome are known: p.Gly11Arg, p.Gly12Arg, p.Asn14Tyr, p.Ser17Phe, p.Ala40Val, p.Asp50Asn, and p.Gly54Glu localized in the N-terminus or in the first extracellular domain (Xu and Nicholson 2013). The mutation p.Asp50Asn of highly conserved aspartic acid in codon 50 responsible for “classical” KID features is identified in most KID patients (Xu and Nicholson 2013). The p.Asp50Asn substitution results from spontaneous methylation and deamination of cysteine in the hypermutable CpG dinucleotide of codon 50.

Although phenotype changes vary over time and manifest different symptoms (even within one family), which results in difficulties in making the correct clinical diagnosis, several patients with p.Asp50Asn mutation in *GJB2* have common leading clinical signs (Table 2). The median age of 25 (out of 26) patients with this mutation presented in the literature is 20 years old (range 10 months to 54 years). In 18 of them, skin manifestation was reported to be present at birth. Hearing impairment developed in all the patients and was designated as profound except for three of them (aged 10, 13, and 42 years old, respectively), in whom it was reported as mild or moderate. Among the skin findings most abundant were: generalized thickened skin, palmoplantar keratoderma, erythematous verrucous plaques, epidermal cysts, and hyperkeratotic lesions (scalp). In 27 % (6/26) of the patients, skin carcinomas were observed, although in the group of adult patients, skin carcinomas were observed in 54 % of patients. Irrespective of age, ocular findings were reported in at least 70 % (18/26) of the patients.

**Table 2:** The phenotypes of all the published patients with p.Asp50Asn mutation in the *GJB2* gene

Sex/age (years)	Ethnicity	Skin abnormalities at birth	Major skin findings	Recurrent skin infections	Hair	Eyelashes/eyebrows	Nail dystrophy	Dental abnormalities	Hearing impairment (bi-/unilateral; severe/moderate)	Ocular signs	Other	References
F/<1	nd	Congenital dermatosis	Dermatosis	nd	Alopecia	nd	+	nd	+ (profound)	Photophobia	nd	Arndt et al. (2010)
F/3	Japanese	nd	Generalized skin dryness with hyperkeratotic plaques on knees, white papules on scalp, angulus oris fissures	nd	Sparse, curly hair	−	+ (middle finger only)	nd	+	Corneal opacity	nd	Yotsumoto et al. (2003)
M/4	Polish	Generally hyperkeratotic skin, especially thick on the back	Generalized thickened skin, especially over joints, ichthyosis-like erythrokeratoderma on cheeks, ears, and chin, reticulated PPK, EVP, deep furrows around the mouth and eyes	+	Stiff and dry	−	−	−	+ (profound)	Photophobia	Acanthosis nigricans in axillary and inner elbow regions and around the nipples, hypohidrosis	This work
F/5	Egyptian	nd	Generalized skin lesions, hyperkeratotic brown color plaques, hypotrichosis	nd	Sparse	Sparse	−	+	+	Corneal opacities	nd	Elsayed et al. (2011)
M/6	French	−	Generalized thickened skin, PPK, EVP	+	Sparse	nd	+	nd	+ (profound)	−	nd	Mazereeuw-Hautier et al. (2007)
F/10	French	Hyperkeratosis (nose)	Generalized thickened skin, PPK, EVP, epidermal cysts, hyperkeratotic lesions (scalp)	−	nd	nd	+	nd	+ (mild)	+	nd	
F/11	Algerian	Erythroderma	Generalized thickened skin, PPK, EVP	−	Sparse	nd	+	nd	+ (profound)	nd	nd	
F/12	UK	Dry and scaly skin, alopecia		+	Alopecia	nd	+	nd	+ (profound)	nd	nd	
M/12	Austrian	nd	PPK, joint contractures of cubita and ankles	nd	Sparse and depigmented	nd	nd	nd	+ (profound)	+	nd	Janecke et al. (2005)
M/13	Japanese	nd	Ichthyosiform eruption, generalized erythrokeratoderma	+	Scarring alopecia	nd	+	nd	+	Vascularizing keratitis	Pannus formation	Yotsumoto et al. (2003)
F/13	nd	−	Diffuse hyperkeratosis, mainly of extremities and external ears	+	nd	nd	nd	nd	+ (mild/moderate)	nd	nd	Janecke et al. (2005)
M/14	Greek	Dry and scaly skin	Generalized thickened skin, PPK, EVP, hyperkeratotic lesions (scalp)	+	Sparse	nd	+	nd	+ (profound)	+	nd	Mazereeuw-Hautier et al. (2007)
F/17	nd	Total scalp alopecia	Keratotic scaling, EVP on hands and feet, PPK with reticulated pattern	nd	Sparse, brittle hair	Xerodermic and cracked eyelids	Brittle toenails with severe dyskeratosis	Delayed eruption	Profound	Active keratitis and blepharitis and photophobia	nd	Alvarez et al. (2003)
F/18	Dutch	−	Thickening and scaling of the skin	nd	Brittle, no pubic and axillary hair	Sparse	+	+	+ (profound)	Keratitis, corneal dystrophy	Spinocellular carcinoma, problems with sweating	van Steensel et al. (2002)
M/21	nd	nd	Generalized thickened skin, PPK, inflammatory nodules, perioral plaques	+	Sparse	−	+ (only toenails)	nd	+	+	−	Gonzalez et al. (2009)
F/23	French	−	Generalized thickened skin, PPK, EVP, epidermal cysts	−	Sparse	nd	+	nd	+ (profound)	−	nd	Mazereeuw-Hautier et al. (2007)
F/30	French	Dry and scaly skin	Generalized thickened skin, PPK, EVP, hyperkeratotic lesions (scalp)	−	Partial	nd	+	nd	+ (profound)	+	Carcinoma	
M/31	German	Present	Spiky hyperkeratosis, sharkskin-like ichthyosis on the face and scalp, generalized hyperkeratosis, and erythroderma	nd	Hypotrichosis	Hypotrichosis	nd	nd	Profound	−	Multiple SCCs since the age of 31 years	van Geel et al. (2002)
F/33	French	nd	Generalized thickened skin, PPK, EVP, inflammatory nodules	+	Alopecia	nd	+	nd	+ (profound)	+	nd	Mazereeuw-Hautier et al. (2007)
F/35	French	Dry and scaly skin		+	Alopecia	nd	+	nd	+ (profound)	+	nd	
M/39	UK	Dry and scaly skin, erythroderma	Generalized thickened skin, PPK, EVP, hyperkeratotic lesions (scalp)	+	Sparse	nd	+	nd	+ (profound)	+	Carcinoma	
F/40	Japanese	nd	Mutilating palmoplantar hyperkeratosis, hypotrichosis	+	nd	nd	+	nd	+	nd	Tumors on the skin of lower limbs and buttocks, mutation in KRT17: c.177C>A	Natsuga et al. (2012)
F/42	French	Dry and scaly skin	Generalized thickened skin, PPK, EVP, epidermal cysts, hyperkeratotic lesions (scalp)	−	Sparse hair	nd	+	nd	+ (profound)	+	Carcinoma	Mazereeuw-Hautier et al. (2007)
F/42	nd	−	Diffuse hyperkeratosis, mainly of extremities	nd	nd	Trichiatic eyelashes	nd	nd	+ (mild/moderate)	+	Sensory neuropathy of the fingers and hands, recurrent axillary and anal fistula	Janecke et al. (2005)
F/54	French	PPK	Generalized thickened skin, PPK, EVP, inflammatory nodules, epidermal cysts	+	Sparse	nd	+	nd	+ (profound)	+	nd	Mazereeuw-Hautier et al. (2007)
nd/nd	nd	nd	Erythrokeratotic cutaneous plaques	nd	nd	nd	nd	nd	nd	nd	SCC	Bergman et al. (2012)

*PPK* palmoplantar keratoderma, *EVP* erythematous verrucous plaques, *nd* no data, “−” not present and “+” present

The degree of difficulty in recognizing KID and in distinguishing the overlapping clinical signs in various skin disorders is well illustrated by the history of patient 1 described in the present study. Over a period of four years from his birth, he had four different clinical diagnoses (X-linked ichthyosis, Netherton syndrome, Menkes syndrome, and ichthyosis again) before the correct diagnosis when KID was finally confirmed.

In the differential diagnostics of KID syndrome, other disorders should also be considered: the classic form of Vohwinkel syndrome (OMIM 124500) with congenital deafness, keratopachydermia, constrictions of fingers and toes, and palmoplantar keratoderma with deafness (OMIM 148350), which are also caused by *GJB2* mutations. Nevertheless, despite the substantial clinical overlap, some differences exist between them as well: keratitis is absent in both of them and keratoderma is limited to the hands, feet, elbows, and knees in the former and to the palms and soles only in the latter syndrome. Furthermore, according to the observation of van Geel et al. (2002) the p.Asp50Asn mutation in *GJB2* can also cause hystrix-like ichthyosis with deafness (HID), where keratitis is not observed either. Such phenotypic discrepancy between KID and HID and the other *GJB2*-caused disorders is poorly understood, but may cause further prolongation of the diagnostic process.

In the case of patient 2, the diagnostic process was shorter, but not very easy, because at the age of 8 months, the patient had only mild clinical expression of the disease—mildly dystrophic nails which could easily have been mistaken for pachyonychia congenita. Immediate correct diagnosis was only possible after clinical examination of the patient’s father, who has, unlike other relatives, severely dysplastic nails, palmoplantar hyperkeratosis, alopecia, and lack of eyebrows and eyelashes. It should be noted, however, that, despite those symptoms, Clouston syndrome had never previously been suggested in the patient’s father. Indeed, only around 30 patients with Clouston syndrome have been published, with very limited phenotypic data available, which makes diagnostics more challenging. Furthermore, several other rare hair–nail ectodermal dysplasias have been identified, which should also be taken into consideration in the diagnostic process, e.g., autosomal recessive “pili torti and onychodysplasia” (OMIM 602032; Calzavara-Pinton et al. 1991), congenital nail dystrophy, hypotrichosis of the scalp with folliculitis decalvans (Barbareschi et al. (1997), and pachyonychia congenita (OMIM 167200). The latter has recently been shown to be falsely recognized in seven patients instead of Clouston syndrome (Hale et al. 2014). Last but not least, KID syndrome diagnosis should also be excluded (van Steensel et al. 2004).

KID and Clouston syndromes are caused by mutation in different connexin genes; however, their symptoms partially overlap (nail dystrophy, hair loss, and palmoplantar keratoderma). This can be due to their function, structure, and ability to cooperate, forming heteromeric (composed of more than one connexin type) connexons.

Connexins belong to the family of gap junction proteins, which enable direct cell-to-cell communication by forming channels in adjacent cells. Hence, they are crucial for maintaining tissue homeostasis, growth control, development, and synchronized response of cell stimuli. Up to now, at least 20 genes of human connexins are known, but skin disorders are only connected with mutations in *GJB2* (Cx26), *GJB6* (Cx30), *GJB3* (Cx31), *GJB4* (Cx30.3), and *GJA1* (Cx43).

Cx26 and Cx30 share a 76 % identity and have a structure typical for connexins: four transmembrane hydrophobic domains, two extracellular highly conserved hydrophilic loops, and three relatively variable cytoplasmic domains (Jan et al. 2004).

Mutations p.Gly11Arg in *GJB6* and p.Asp50Asn in *GJB2* are located in the N-terminus of Cx30 and in the first extracellular domain of Cx26, respectively. Both domains face pore channels and, thus, mutations in these regions can lead to changes in connexon conductance or even loss of channel function (for details, see Oshima et al. 2011, Levit et al. 2012, and Essenfelder et al. 2004).

While the *GJB2* gene mutations associated with skin symptoms all cause deafness, mutations in the *GJB6* gene result in skin disease, usually without hearing impairment (van Steensel et al. 2004). This can be explained by connexin redundancy. In the inner ear, the wild type of connexin 26 can compensate for the lack of activity of mutated connexin 30, but not in the skin. Such compensation is not possible in the opposite direction, either in the skin or in the inner ear. The key to clarifying this phenomenon is different permeability for ions for both the homotypic (composed of one connexin type) and heterotypic (composed of more than one connexin type) connexons. Therefore, mutated Cx26 exerts a dominant negative effect on the wild type of other co-expressed connexins, like, for example, on Cx30 in the skin. Cohen-Salmon et al. (2002) concluded that epithelial gap junctions containing Cx26 are essential for the cochlear function and cell survival. This observation and explanation is complicated by the finding of Jan et al. (2004), who described a case of KID syndrome with sensorineural hearing loss and congenital atrichia caused by mutation in the gene *GJB6* (Cx30), which is usually connected with Clouston syndrome. Similarly, Sugiura et al. (2013) described a patient with Clouston syndrome and sensorineural hearing loss and photophobia, but in this patient, apart from mutation in the *GJB6* gene, the *GJB2* gene variant was found. Another patient with a phenotype resembling Clouston syndrome but with deafness and mutation in the *GJB2* gene was described by van Steensel et al. (2004). To establish a genotype–phenotype correlation in gap junction skin syndromes, further studies and observations are needed. New gap junction syndromes of the skin are still being described, which can have a great significance for establishing this correlation (de Zwart-Storm et al. 2011).

It seems that there are still some unrecognized disorders of the skin to be discovered by inquisitive geneticists and dermatologists, who should also clinically evaluate the pedigree of family members and verify clinical recognition using molecular techniques. Similarly, the genetic background of several clinically distinguished disorders, including around 70 % of about 170–200 known ectodermal dysplasias, still lacks genetic explanation (Visinoni et al. 2009; Irvine 2009).

The conclusion was drawn that the proper diagnosis of gap junction syndromic skin disorders is still a challenge, especially due to the extremely low incidence of this disorder [no patient of Polish origin with Clouston syndrome and only one with KID-like syndrome (de Zwart-Storm et al. 2011) had been described in the literature before]. However, it can be neither neglected nor underestimated due to variable prognosis and highly differing health complications that may occur (like SCC in KID syndrome).

## References

[CR1] Alvarez A, del Castillo I, Pera A, Villamar M, Moreno-Pelayo MA, Moreno F, Moreno R, Tapia MC (2003). De novo mutation in the gene encoding connexin-26 (GJB2) in a sporadic case of keratitis–ichthyosis–deafness (KID) syndrome. Am J Med Genet A.

[CR2] Arndt S, Aschendorff A, Schild C, Beck R, Maier W, Laszig R, Birkenhäger R (2010). A novel dominant and a de novo mutation in the GJB2 gene (connexin-26) cause keratitis–ichthyosis–deafness syndrome: implication for cochlear implantation. Otol Neurotol.

[CR3] Avshalumova L, Fabrikant J, Koriakos A (2014). Overview of skin diseases linked to connexin gene mutations. Int J Dermatol.

[CR4] Barbareschi M, Cambiaghi S, Crupi AC, Tadini G (1997). Family with “pure” hair–nail ectodermal dysplasia. Am J Med Genet.

[CR5] Bergman R, Mercer A, Indelman M, Sprecher E, Haim N, Zoller L, Ben-Izhak O, Hershkovitz D (2012). KID syndrome: histopathological, immunohistochemical and molecular analysis of precancerous and cancerous skin lesions. Br J Dermatol.

[CR6] Burns FS (1915). A case of generalized congenital keratoderma with unusual involvement of the eyes, ears, and nasal and buccous membranes. J Cutan Dis.

[CR7] Caceres-Rios H, Tamayo-Sanchez L, Duran-Mckinster C, de la Luz Orozco M, Ruiz-Maldonado R (1996) Keratitis, ichthyosis, and deafness (KID syndrome): review of the literature and proposal of a new terminology. Pediatr Dermatol 13:105–113 10.1111/j.1525-1470.1996.tb01414.x 9122065

[CR8] Calzavara-Pinton P, Carlino A, Benetti A, De Panfilis G (1991). Pili torti and onychodysplasia. Report of a previously undescribed hidrotic ectodermal dysplasia. Dermatologica.

[CR9] Cohen-Salmon M, Ott T, Michel V, Hardelin JP, Perfettini I, Eybalin M, Wu T, Marcus DC, Wangemann P, Willecke K, Petit C (2002). Targeted ablation of connexin26 in the inner ear epithelial gap junction network causes hearing impairment and cell death. Curr Biol.

[CR10] de Zwart-Storm EA, Rosa RF, Martin PE, Foelster-Holst R, Frank J, Bau AE, Zen PR, Graziadio C, Paskulin GA, Kamps MA, van Geel M, van Steensel MA (2011). Molecular analysis of connexin26 asparagine14 mutations associated with syndromic skin phenotypes. Exp Dermatol.

[CR11] Elsayed SM, Seifeldeen NS, Bolz H (2011). Connexin 26 (GJB2) mutation in KID syndrome: an Egyptian patient. Egypt J Med Hum Genet.

[CR12] Essenfelder GM, Bruzzone R, Lamartine J, Charollais A, Blanchet-Bardon C, Barbe MT, Meda P, Waksman G (2004). Connexin30 mutations responsible for hidrotic ectodermal dysplasia cause abnormal hemichannel activity. Hum Mol Genet.

[CR13] Gilliam A, Williams ML (2002). Fatal septicemia in an infant with keratitis, ichthyosis, and deafness (KID) syndrome. Pediatr Dermatol.

[CR14] Gómez-Faiña P, Ruiz-Viñals AT, Buil-Calvo JA, España-Albelda A, Pazos-López M, Castilla-Céspedes M (2006). Paciente con enfermedad corneal severa en el context del syndrome de KID [Patient with severe corneal disease in KID syndrome]. Arch Soc Esp Oftalmol.

[CR15] Gonzalez ME, Tlougan BE, Price HN, Patel R, Kamino H, Schaffer JV (2009). Keratitis–ichthyosis–deafness (KID) syndrome. Dermatol Online J.

[CR16] Hale GI, Wilson NJ, Smith FJ, Wylie G, Schwartz ME, Zamiri M (2014). Mutations in GJB6 causing phenotype resembling pachyonychia congenita. Br J Dermatol.

[CR17] Hassed SJ, Kincannon JM, Arnold GL (1996). Clouston syndrome: an ectodermal dysplasia without significant dental findings. Am J Med Genet.

[CR18] Hazen PG, Carney P, Lynch WS (1989). Keratitis, ichthyosis, and deafness syndrome with development of multiple cutaneous neoplasms. Int J Dermatol.

[CR19] Irvine AD (2009). Towards a unified classification of the ectodermal dysplasias: opportunities outweigh challenges. Am J Med Genet Part A.

[CR20] Jan AY, Amin S, Ratajczak P, Richard G, Sybert VP (2004). Genetic heterogeneity of KID syndrome: identification of a Cx30 gene (GJB6) mutation in a patient with KID syndrome and congenital atrichia. J Invest Dermatol.

[CR21] Janecke AR, Hennies HC, Günther B, Gansl G, Smolle J, Messmer EM, Utermann G, Rittinger O (2005). GJB2 mutations in keratitis–ichthyosis–deafness syndrome including its fatal form. Am J Med Genet A.

[CR22] Kibar Z, Der Kaloustian VM, Brais B, Hani V, Fraser FC, Rouleau GA (1996). The gene responsible for Clouston hidrotic ectodermal dysplasia maps to the pericentromeric region of chromosome 13q. Hum Mol Genet.

[CR23] Levit NA, Mese G, Basaly MG, White TW (2012). Pathological hemichannels associated with human Cx26 mutations causing Keratitis–Ichthyosis–Deafness syndrome. Biochim Biophys Acta.

[CR24] Mazereeuw-Hautier J, Bitoun E, Chevrant-Breton J, Man SY, Bodemer C, Prins C, Antille C, Saurat JH, Atherton D, Harper JI, Kelsell DP, Hovnanian A (2007). Keratitis–ichthyosis–deafness syndrome: disease expression and spectrum of connexin 26 (GJB2) mutations in 14 patients. Br J Dermatol.

[CR25] Natsuga K, Shinkuma S, Kanda M, Suzuki Y, Chosa N, Narita Y, Setoyama M, Nishie W, Akiyama M, Shimizu H (2012). Possible modifier effects of keratin 17 gene mutation on keratitis–ichthyosis–deafness syndrome. J Br J Dermatol.

[CR26] Oshima A, Tani K, Toloue MM, Hiroaki Y, Smock A, Inukai S, Cone A, Nicholson BJ, Sosinsky GE, Fujiyoshi Y (2011). Asymmetric configurations and N-terminal rearrangements in connexin26 gap junction channels. J Mol Biol.

[CR27] Skinner BA, Greist MC, Norins AL (1981). The keratitis, ichthyosis, and deafness (KID) syndrome. Arch Dermatol.

[CR28] Sugiura K, Teranishi M, Matsumoto Y, Akiyama M (2013). Clouston syndrome with heterozygous GJB6 mutation p.Ala88Val and GJB2 variant p.Val27Ile revealing mild sensorineural hearing loss and photophobia. JAMA Dermatol.

[CR29] van Geel M, van Steensel MA, Küster W, Hennies HC, Happle R, Steijlen PM, König A (2002). HID and KID syndromes are associated with the same connexin 26 mutation. Br J Dermatol.

[CR30] van Steensel MA, van Geel M, Nahuys M, Henk Sillevis Smitt J, Steijlen PM (2002). A novel connexin 26 mutation in a patient diagnosed with keratitis–ichthyosis–deafness syndrome. J Invest Dermatol.

[CR31] van Steensel MA, Steijlen PM, Bladergroen RS, Hoefsloot EH, van Ravenswaaij-Arts CM, van Geel M (2004). A phenotype resembling the Clouston syndrome with deafness is associated with a novel missense GJB2 mutation. J Invest Dermatol.

[CR32] Visinoni ÁF, Lisboa-Costa T, Pagnan NAB, Chautard-Freire-Maia EA (2009). Ectodermal dysplasias: clinical and molecular review. Am J Med Genet Part A.

[CR33] Xu J, Nicholson BJ (2013). The role of connexins in ear and skin physiology—functional insights from disease-associated mutations. Biochim Biophys Acta.

[CR34] Yotsumoto S, Hashiguchi T, Chen X, Ohtake N, Tomitaka A, Akamatsu H, Matsunaga K, Shiraishi S, Miura H, Adachi J, Kanzaki T (2003). Novel mutations in GJB2 encoding connexin-26 in Japanese patients with keratitis–ichthyosis–deafness syndrome. Br J Dermatol.

